# Long-Term Follow-Up of Three Family Members with a Novel *NNT* Pathogenic Variant Causing Primary Adrenal Insufficiency

**DOI:** 10.3390/genes13050717

**Published:** 2022-04-20

**Authors:** Tjasa Krasovec, Jaka Sikonja, Mojca Zerjav Tansek, Marusa Debeljak, Sasa Ilovar, Katarina Trebusak Podkrajsek, Sara Bertok, Tine Tesovnik, Jernej Kovac, Jasna Suput Omladic, Michaela F. Hartmann, Stefan A. Wudy, Magdalena Avbelj Stefanija, Tadej Battelino, Primoz Kotnik, Urh Groselj

**Affiliations:** 1 Faculty of Medicine, University of Ljubljana, Vrazov Trg 2, SI-1000 Ljubljana, Slovenia; krasovec.tjasa@gmail.com (T.K.); jaka.sikonja@gmail.com (J.S.); mojca.zerjav-tansek@mf.uni-lj.si (M.Z.T.); katarina.trebusakpodkrajsek@mf.uni-lj.si (K.T.P.); jernej.kovac@kclj.si (J.K.); jasna.suputomladic@kclj.si (J.S.O.); magdalena.avbelj@mf.uni-lj.si (M.A.S.); tadej.battelino@mf.uni-lj.si (T.B.); primoz.kotnik@mf.uni-lj.si (P.K.); 2 Department of Endocrinology, Diabetes, and Metabolic Diseases, University Children’s Hospital, University Medical Centre Ljubljana, Bohoriceva Ulica 20, SI-1000 Ljubljana, Slovenia; sara.bertok@kclj.si; 3 Clinical Institute of Special Laboratory Diagnostics, University Children’s Hospital, University Medical Centre Ljubljana, Vrazov Trg 1, SI-1000 Ljubljana, Slovenia; marusa.debeljak@kclj.si (M.D.); tine.tesovnik@kclj.si (T.T.); 4 Department of Pediatric Cardiology, University Children’s Hospital, University Medical Centre Ljubljana, Bohoriceva Ulica 20, SI-1000 Ljubljana, Slovenia; sasa.ilovar@kclj.si; 5 Laboratory for Translational Hormone Analytics, Steroid Research and Mass Spectrometry Unit, Division of Pediatric Endocrinology and Diabetology, Center of Child and Adolescent Medicine, Justus Liebig University, Feulgenstraße 12, 35392 Giessen, Germany; michaela.hartmann@paediat.med.uni-giessen.de (M.F.H.); stefan.wudy@paediat.med.uni-giessen.de (S.A.W.)

**Keywords:** nicotinamide nucleotide transhydrogenase, NNT deficiency, primary adrenal insufficiency, nucleotide duplication, hearth sonography, pubertal development, testicular volume, bone density

## Abstract

Nicotinamide nucleotide transhydrogenase (NNT) deficiency causes primary adrenal insufficiency (PAI) and possibly some extra-adrenal manifestations. A limited number of these patients were previously described. We present the clinical and genetic characteristics of three family members with a biallelic novel pathogenic variant in the *NNT* gene. The patients were followed until the ages of 21.6, 20.2, and 4.2 years. PAI was diagnosed in the eldest two brothers after an Addisonian crisis and the third was diagnosed at the age of 4.5 months in the asymptomatic stage due to the genetic screening of family members. Whole exome sequencing with a targeted interpretation of variants in genes related to PAI was performed in all the patients. The urinary steroid metabolome was determined by gas chromatography–mass spectrometry in the asymptomatic patient. The three patients, who were homozygous for c.1575dup in the *NNT* gene, developed isolated glucocorticoid deficiency. The urinary steroid metabolome showed normal excretion of cortisol metabolites. The adolescent patients had slow pubertal progression with low–normal testicular volume, while testicular endocrine function was normal. Bone mineral density was in the range for osteopenia in both grown-up siblings. Echocardiography revealed no structural or functional heart abnormalities. This article is among the first with a comprehensive and chronologically-detailed description of patients with NNT deficiency.

## 1. Introduction

Primary adrenal insufficiency (PAI) is a life-threatening condition that is characterized by the deficient production of glucocorticoids and/or mineralocorticoids due to the unresponsive or dysfunctional adrenocortical tissue. The incidence of PAI is estimated at around 5 cases per million in white adults and is even less common in the pediatric population [1,2,3,4,5].

In children, most causes of PAI are of genetic origin, with congenital adrenal hyperplasia (CAH) being the most common [1,2,5,6]. Other inherited etiologies are adrenal gland developmental disorders, Triple A syndrome, metabolic causes, and corticotropin (ACTH) resistance, including familial glucocorticoid deficiency (FGD) [1,7]. Autoimmune processes, infections, infiltrative diseases, adrenal hemorrhage, bilateral adrenalectomy, and some medications can cause non-genetic PAI [1].

The clinical signs of PAI include hyperpigmentation of the skin and mucous membranes in addition to hypoglycemia, salt wasting, hypotension, and other non-specific symptoms, such as fatigue, weight loss, failure to thrive, depression, and convulsions [1,2,4,7]. The median age at diagnosis of PAI in the tertiary pediatric endocrinology department is 1.5 years [8]. Treatment centers around the replacement of glucocorticoids and mineralocorticoids to maintain a physiologic water–electrolyte homeostasis together with attainment of normal physical and pubertal growth [7].

Nicotinamide nucleotide transhydrogenase (NNT) is an integral protein of the inner mitochondrial membrane that generates reduced nicotinamide adenine dinucleotide phosphate (NADPH) using the energy from the mitochondrial proton gradient [2,9,10,11,12]. NADPH is an essential factor in steroidogenesis, and it is needed to maintain the cellular balance of reactive oxygen species (ROS) through the glutathione peroxidase pathway [2,9,10,11,12]. The *NNT* gene was discovered as a causative gene for FGD [12]. It is ubiquitously expressed in the human body, and, furthermore, its highest levels were observed in the adrenals, bladder, heart, kidney, thyroid, and adipose tissue, thereby suggesting that extra-adrenal manifestations, such as cardiomyopathy and hypothyroidism, could occur in these patients in addition to PAI [9,12].

To date, around 50 NNT-deficient patients were described in the literature with scarce data on long-term follow-up and management. We present the detailed, long-term clinical characteristics of three Slovenian siblings with PAI due to a disease-causing variant in the *NNT* gene.

## 2. Materials and Methods

We performed a retrospective evaluation of the clinical management of three brothers who were followed at the University Children’s Hospital, University Medical Centre Ljubljana up until the ages of 21.6, 20.2, and 4.2 years. The two oldest siblings (patients 1 and 2) were diagnosed at the age of 1.4 and 1.1 years, respectively, after an acute adrenal crisis. The youngest brother (patient 3) was diagnosed at the age of 4.5 months in the asymptomatic phase. We also performed a clinical investigation and genetic testing on both of their parents.

UK-WHO growth charts were used to calculate height, weight, and body mass index (BMI) percentiles/Z scores. Body surface area was calculated with Haycock’s formula [13]. The pubertal stages were determined according to Tanner classification. The testicular volume was measured by an orchidometer. The laboratory measurements of hormones were performed from morning blood samples and analyzed by standard methods. Bone mineral density (BMD) was measured by dual-energy X-ray absorptiometry (DEXA). Testicular and thyroid ultrasonography, echocardiography, and electrocardiogram (ECG) were performed and interpreted by respective specialists. Diagnosis of PAI was made according to the following criteria [14]:

1. hypocortisolism (morning serum cortisol levels <140 nmol/L) coupled with elevated ACTH levels (>2-times above the upper limit of normal)
2. insufficient response of adrenal glands at ACTH stimulation test (serum cortisol levels <500 nmol/L at 30 or 60 min after intravenous administration of ACTH in a standard dose of 15 μg/kg for infants and 125 μg for children <2 years of age).

To investigate gonadal function, we performed a gonadotropin-releasing hormone (GnRH) stimulation test and measured the luteinizing hormone (LH) and follicle stimulating hormone (FSH) levels before (basal levels) and 20 min, 30 min, and 60 min after administration of 100 μg GnRH. The LH and FSH levels were measured by immunochemiluminometric assay, and the results were interpreted according to the reference intervals, which were reported by Resende et al. [15].

In patient 3, a gas chromatography–mass spectrometry (GC-MS) urinary steroid metabolome analysis was made from spot urine when the patient was 6 months of age and still untreated. In brief, free and conjugated urinary steroids were extracted by solid-phase extraction and the conjugates were enzymatically hydrolyzed. The hydrolyzed steroids were re-extracted, and internal standards were added. An aliquot of the derivatized extract (methyloxime-trimethylsilyl ethers) was subjected to GC-MS analysis [16].

The genomic DNA was isolated from peripheral blood samples with the FlexiGene DNA Kit 250 (Qiagen, Hilden, Germany). Whole exome sequencing (WES) was performed on all three patients using the Agilet SureSelect Human AllExon V5 kit for whole exome enrichment and Illumina NovaSeq 6000 (Illumina, San Diego, CA, USA). Genetic variants with a coverage >15× were filtered with VarAft software after a bcbio-nextgen toolkit bioinformatics analysis [17,18]. We evaluated rare variants with minor allele frequencies (less than 5%) in a wider panel of genes, which were reported to be related to adrenal insufficiency (*AAAS*, *ABCC8*, *ABCD1*, *AIP*, *AIRE*, *AKT1*, *BAP1*, *BCOR*, *BRAF*, *CDH23*, *CTNNB1*, *CYP11A1*, *CYP11B1*, *CYP11B2*, *CYP17A1*, *DHX37*, *GATA4*, *GATB*, *GATC*, *GK*, *GLI3*, *GMPPA*, *HBB*, *HERC2*, *HSD11B2*, *HSD17B4*, *HSD3B2*, *IARS2*, *IPW*, *KANSL1*, *KCNJ11*, *LHX4*, *LIPA*, *MAGEL2*, *MAP3K1*, *MC2R*, *MCM4*, *MEN1*, *MKRN3*, *MRAP*, *MRPS25*, *MRPS7*, *NDN*, *NF2*, *NFKB2*, *NNT*, *NPAP1*, *NR0B1*, *NR3C1*, *NR3C2*, *NR5A1*, *OCA2*, *PCSK1*, *PDGFB*, *PEX1*, *PEX10*, *PEX11B*, *PEX12*, *PEX13*, *PEX14*, *PEX16*, *PEX19*, *PEX2*, *PEX26*, *PEX3*, *PEX5*, *PEX6*, *PIK3CA*, *POMC*, *POR*, *PROP1*, *PWAR1*, *PWRN1*, *QRSL1*, *RBM28*, *SAMD9*, *SCNN1B*, *SGPL1*, *SMARCB1*, *SMARCE1*, *SMO*, *SNORD115-1*, *SNORD116-1*, *SNRPN*, *SOX9*, *SRY*, *STAR*, *STEAP3*, *SUFU*, *TBCK*, *TBX19*, *TCTN3*, *TERT*, *TRAPPC11*, *TXNRD2*, *WT1*, *WWOX*, *ZFPM2*) [19].

All clinical data were acquired from electronic medical records and Microsoft Office 365 (Microsoft Corporation, Redmond, WA, USA) was used for data collection, analysis, and visualization.

## 3. Results

### 3.1. General Clinical Characteristics

The three brothers were born with normal body weight and body height after three uneventful pregnancies. In the eldest and youngest sibling, vaginal delivery was triggered 1 week before term due to placental insufficiency. None of them showed signs of adrenal insufficiency in the neonatal period; hypoglycemia was not reported, and all three siblings had neonatal indirect hyperbilirubinemia, which resolved with phototherapy. In the second year, patient 1 already had notably hyperpigmented skin and was presented to the emergency department in epileptic status that resolved only after pharmacological intervention and was followed by cardiac arrest. After a successful resuscitation, initial investigation revealed that patient 1 had pneumonia, severe hypoglycemia (5.4 mg/dL; reference range (RF): 65–110 mg/dL), hypocortisolism (6 nmol/L; RF: 140–690 nmol/L), and high ACTH levels (678 pmol/L; RF: <10.2 pmol/L). Similarly, parents reported increasing pigmentation, particularly around the scrotum, knees, and neck in patient 2, who presented in a febrile and fatigued state at the age of 1.1 years. PAI was diagnosed from the decreased cortisol levels (53 nmol/L; RF: 140–690 nmol/L) and markedly increased ACTH (905 pmol/L; RF: <10.2 pmol/L). Acute presentations incited the testing of patient 3 who was diagnosed in the asymptomatic phase at 4.5 months of age. Non-provoked cortisol levels were in the normal range (276 nmol/L; RF: 140–690 nmol/L) together with elevated ACTH (54.4 pmol/L; RF: <10.2 pmol/L). An additional ACTH stimulation test revealed the insufficient response of the adrenal glands to ACTH, as peak cortisol levels remained below 500 nmol/L.

**Figure 1:**
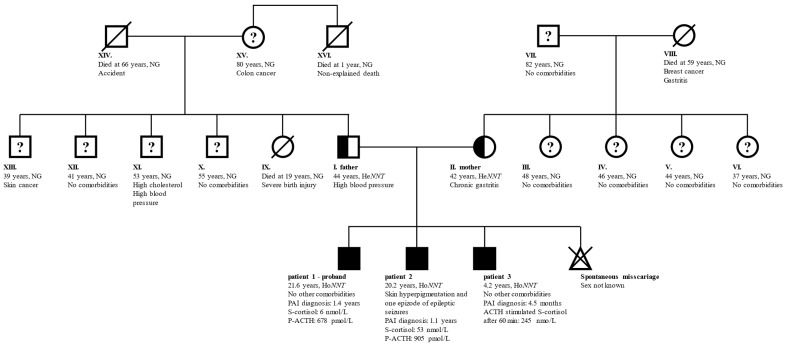
**General characteristics of family members.** Full symbols indicate patients with confirmed PAI, half-filled symbols confirmed carriers of pathogenic *NNT* variant, and “?” family members without PAI, but no information on carrier status. Family member indexes, age at last follow-up, relevant comorbidities, genetic status, the age at diagnosis of PAI, and laboratory results indicative of PAI are written. Patients 1 and 2 presented with an acute adrenal crisis and as probands drove the early identification of PAI in patient 3. Children of members III–VI and IX–XIII were not affected and are therefore excluded from the genogram. Member XVI died at the age of 1 year from an unidentified cause. Abbreviations: ACTH—corticotropin; *He/HoNNT*—heterozygous/homozygous for NM_182977:c.1575dupA in *NNT*; NG—genetic testing for *NNT* variant not performed; *NNT*—gene for nicotinamide nucleotide transhydrogenase; P—plasma; PAI—primary adrenal insufficiency; S—serum.

The parents of the three children were in good general health without signs of PAI. The father had mildly elevated arterial blood pressure and the mother had chronic gastritis due to an H. pylori infection. None of the two reported heart, thyroid, gonadal, or endocrine diseases. The couple did not encounter problems at the conception of their children, however, the mother reported one spontaneous miscarriage. While the parents did not report consanguinity, both their families originated from the same local environment in central Slovenia. An unexplained death occurred with the father’s uncle who died at the age of 1 year. None of the other family members was diagnosed with PAI. A family genogram with the general characteristics of the family members is presented in Figure 1.

**Figure 2:**
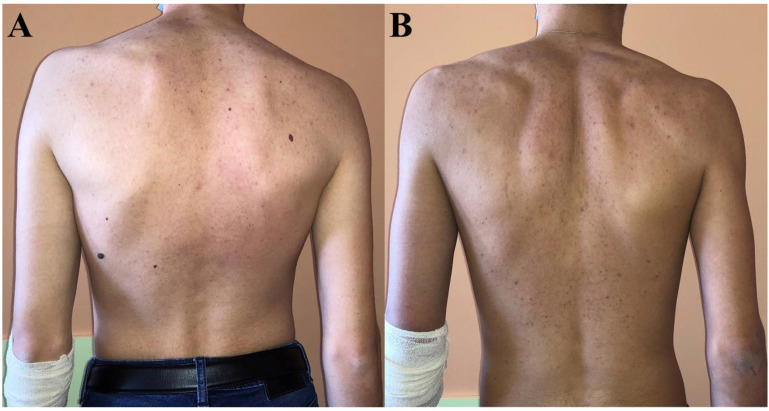
**Skin coloration at last visit in patient 1 (A) and patient 2 (B).** Patient 2 was significantly more hyperpigmented—particularly around elbows—than patient 1 who had the same skin tone as their parents and patient 3.

While no skin changes were observed in patient 3, hyperpigmentation completely resolved in patient 1 after treatment initiation, but not in patient 2 in whom it had persisted even up to the last visit (Figure 2).

**Figure 3:**
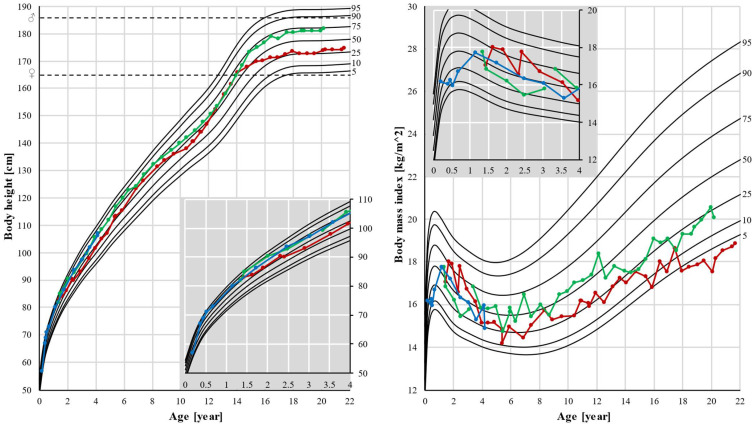
**Body height and body mass index (BMI).** Pubertal growth spurt was earlier in patient 1, and also growth stopped earlier in this patient, resulting in a lower final height. Black lines represent the 5th, 10th, 25th, 50th, 75th, 90th, and 95th percentiles. Dots represent individual measurements: red for patient 1, green for patient 2, and blue for patient 3. Dashed lines represent final heights of the father (♂) and mother (♀). In the corner, there is a magnified part of the graph up to age 4.

Patients 1 and 2 had normal body height as their height followed their genetic potential. Normal body weight and BMI were observed in patient 2, but a significant decrease in weight and BMI was seen in patient 1. After the age of 15, his BMI decreased from the 25th percentile to below the 5th percentile (Figure 3).

Mineralocorticoid function accessed by electrolyte levels, aldosterone, and plasma renin activity was unimpaired in all three brothers. Thyroid endocrine function was normal and thyroid ultrasound revealed normal gland volume and no structural abnormalities.

### 3.2. Genetics

WES analysis identified a homozygous duplication of one nucleotide NM_182977:c.1575dup in the *NNT* gene in all three patients. The variant could cause defective enzyme activity due to a frameshift (p.Pro526ThrfsTer65) that introduced a premature stop codon. Sanger sequencing-based segregation analysis confirmed the presence of this variant in the homozygous state in all the patients and in the heterozygous state in their healthy parents.

The detected variant fits in an autosomal recessive model of inheritance in the family. Its pathogenicity was predicted through in silico programs: CADD (Combined Annotation Dependent Depletion) score (35) and MutationTaster (disease causing 0.999). The variant was not reported in the dbSNP or gnomAD database. According to the ACMG criteria, it was classified as pathogenic with the following grades (PVS1, PM2, PP5).

### 3.3. Hormone Replacement Therapy and Monitoring

**Figure 4:**
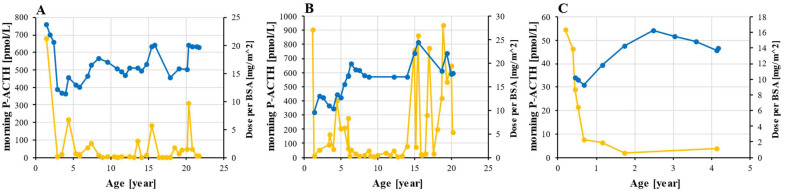
**Hydrocortisone dosing and ACTH monitoring.** ACTH (orange) was measured from morning plasma sample and hydrocortisone dose (blue) was adjusted to BSA in patient 1 (**A**), 2 (**B**), and 3 (**C**). Abbreviations: ACTH—corticotropin; BSA—body surface area; P—plasma.

At the time of PAI diagnosis, all three patients had been started on a glucocorticoid supplementation with oral hydrocortisone, which was followed by a significant decrease in ACTH levels. The dynamics of ACTH and a daily hydrocortisone dose are shown in Figure 4. Hormone supplementation was uninterrupted throughout the follow-up and very few doses were missed as the parents strictly monitored the patients in childhood and given that patients 1 and 2 experienced fatigue when they missed a dose. The average dose of hydrocortisone was 16.6 mg/m^2^ for patients 1 and 2 and 13.0 mg/m^2^ for patient 3. In all three, the minimal prescribed dose was 9.3 mg/m^2^ and the maximum was 24.5 mg/m^2^. The period of optimal ACTH levels in patient 2 between the ages of 7 and 15 were followed by a dramatic increase in ACTH with several values exceeding 500 pmol/L. According to the patient, this was not a result of non-adherence to the therapy.

### 3.4. Sex Maturation

Patient 2 had inguinal retention of one testicle at the age of 8.9 years, which was surgically corrected. The age at onset of first pubertal changes in both patient 1 and patient 2 were in the normal range for boys (11–12 years), however, their testicular volume did not rise as expected. The testicular volume of patient 2 was 15 mL at the age of 17.5 years and 10–12 mL in patient 1 at 17.9 years. The measurements at the last visit revealed equally large testes and a testicular volume of 15 mL in both patients, with normal testosterone (25–30 nmol/L; RF: 12–42 nmol/L) and inhibin B levels (200 ng/L; RF: 25–325 ng/L). Basal as well as stimulated LH and FSH values 20 min, 30 min, and 60 min after GnRH administration were normal for their pubertal stage. Moreover, adrenal androgens – androstenedione (average pubertal values in patients 1 and 2: 0.87 and 1.14 nmol/L; RF: 0.7–10.1 nmol/L) and DHEA-S (average pubertal values: 0.76 and 0.44 μmol/L; RF: 3.6–12.9 μmol/L) were low–normal throughout puberty. In addition, 17-hydroxyprogesterone levels were normal.

Both patients 1 and 2 developed a unilateral varicocele and underwent embolization surgery at the age of 18.5 and 17.4 years, respectively. A routine ultrasound of the testicles did not confirm any tumor-like formations.

The GC-MS urinary steroid metabolome analysis (Table 1) at the age of 6 months in patient 3 revealed normal excretion of cortisol and corticosterone, as well as DHEA metabolites. Thus, inborn errors from cortisol biosynthesis such as StAR-defect, P450 side chain cleavage defect, 3β-hydroxysteroid dehydrogenase deficiency, 21-hydroxylase deficiency, 11-hydroxylase deficiency, 17-hydroxylase/17,20 lyase deficiency, and Smith Lemli Opitz syndrome could be excluded.

### 3.5. Bone Mineral Density

The first DEXA measurement was performed in patients 1 and 2 at the age of 13.6 and 14.4 years and revealed markedly lowered BMD in the range of osteopenia from the whole-body scan and in lumbar L1–L4 segment. The BMD in patient 1 decreased over the course of follow-up. During the last visit from patient 1, the whole body and L1–L4 Z scores were –2.4 and –2.5, respectively. On the other hand, patient 2 experienced an improvement in the BMD of the whole body and L1–4 Z scores during the last visit. The values were –1.8 and –1.0, respectively. However, patient 2 had a forearm fracture due to a high-energy trauma at the age of 12 years. In the presence of low 25-OH-vitamin D (33 and 35 nmol/L; RF: 32–165 nmol/L) and normal calcium levels in patients 1 and 2, vitamin D and calcium supplementation were started at the age of 13.6 and 12.1 years, respectively, and continued through the follow-up.

### 3.6. Heart Function

All three patients were clinically without symptoms related to heart disease. ECG and transthoracic echo were performed in all three patients, and we did not find any pathological changes in the structure or function of the heart.

A slightly rightwards heart axis was noticed on the ECG of all three patients (92–120°). The rhythm was sinus, the length of QRS was normal (79–100 ms), and no repolarization abnormalities were present.

On echo, the size of the left ventricle (LV) was normal in all three patients (end diastolic diameter of LV (LVDd) in patient 1 was 4.8 cm, in patient 2 was 5.0 cm, and in patient 3 was 3.2 cm (RF: 4.2–5.8 cm for patients 1 and 2, and 3.0–4.2 cm for patient 3)). The LV wall thickness was within the normal range (thickness of the interventricular septum in diastole (IVSd) was 0.8 cm, 0.9 cm, and 0.6 cm, while the thickness of the posterior wall of the LV (LVPWd) was 0.7 cm, 0.8 cm, and 0.5 cm, respectively (RF for patients 1 and 2: 0.6–1.0 cm; RF for patient 3: 0.4–0.7 cm)). The systolic as well as diastolic function of LV in all three patients were normal (EF LV 69–70%; RF: >52%). No apparent hypertrabeculation of the LV was noticed. The function, size, and wall thickness of the right ventricle was normal in all three.

## 4. Discussion

We described the detailed, long-term follow-up of three siblings carrying a novel disease-causing variant in the *NNT* gene that is associated with an isolated glucocorticoid deficiency.

Two patients presented with an Addisonian crisis to the emergency department in the second year of life. The acute states were triggered by an infection and were preceded by hyperpigmentation of the skin. The age at presentation in patients with *NNT* disease-causing variants, which is reported in the literature, was around 12 months—with a wide range from 3 days to 4 years [9,20]. Other authors stated that a stressful event (e.g., infection) and concomitant mineralocorticoid deficiency probably led to an earlier manifestation of the disease [9]. In our case series, basal cortisol levels were lower with a higher age at diagnosis and the diagnosis of PAI in the youngest patient, who was 4.5 months of age, was done only after an ACTH stimulation test. All the presented patients had no PAI-related symptoms in the neonatal period. However, hyperpigmentation was observed before the acute presentation, and it was reported to be a common first sign of the disease [21]. To prevent the acute manifestation of the disease, as was the case in the youngest patient in our study, we advise that siblings of patients with confirmed biallelic *NNT*-related PAI be genetically tested in the first months of life and evaluated for PAI, especially if hyperpigmentation occurs.

In animal and laboratory studies, mice with defective *NNT* experienced excessive cell apoptosis in the adrenal gland zona fasciculata, and an increase of ROS was observed in the human adrenocortical H295R cell line [12]. In the 6-month-old patient 3, a GC-MS urinary steroid metabolome analysis showed a normal basal excretion of cortisol metabolites. However, plasma ACTH was already elevated. Taken together, these data suggest that adrenal disease and the impairment of steroidogenesis in NNT deficiency are gradually progressing processes. We speculate that, at first, functioning glucocorticoid-producing cells produced sufficient amounts of cortisol. With ongoing cell apoptosis there will be less of these cells and, subsequently, PAI will develop. *NNT* is expressed in various tissues in the human body. Although no firm evidence exists, one proposed underlying mechanism is that NNT plays an essential role in ROS detoxification in the adrenal glands, and other body tissues are not primarily affected due to a compensation from antioxidative pathways other than the NADPH-glutathione pathway [12,21]. Furthermore, an alternative explanation is that ACTH stimulation of the adrenals, due to a high cortisol demand, results in the acceleration of steroidogenesis and in the associated increased production of ROS, which overwhelms the antioxidant systems in the adrenals. Other tissues are possibly not affected because their ROS generation may not reach such extreme levels.

We report a novel single codon duplication (NM_182977:c.1575dup) in the *NNT* gene, which was found in a family with PAI. The variant is located in the membrane spanning domain in exon 11, which causes a frameshift and introduces an early stop codon that likely resulted in a defective protein. According to the Human Gene Mutation Database, 47 different disease-causing variants were previously described: 26 missense/nonsense variants, 11 deletions, 7 duplications, and 3 splice site variants.

The parents were heterozygous for the variant and reported no NNT deficiency-related symptoms. Since marriages between individuals from the same local environment were even more prevalent a few decades ago, it is possible that the uncle of the father (family member XVI in Figure 1) who died at the age of 1 year from an unidentified cause was also homozygous for this *NNT* variant and died due to an Addisonian crisis. Unfortunately, this hypothesis was impossible to confirm.

The patients and their parents reported uninterrupted hormone supplementation and feelings of fatigue after a missed dose. However, there is still a possibility of non-adherence to prescribed therapy as persistent increases in hydrocortisone doses did not result in a decrease in the ACTH concentration in patient 2. Another explanation for significant ACTH elevations, which were indirectly manifested by the increased pigmentation of his skin, could be an autonomous ACTH-secreting tissue. Similar cases, although rare, are described in the literature, where patients with PAI after several decades of hormone replacement therapy developed increasing skin pigmentation and had a progressive rise of ACTH and an absence of the diurnal rhythm of ACTH. The evaluation with magnetic resonance imaging then revealed pituitary hyperplasia and, in some cases, a pituitary adenoma [22,23,24]. Further follow-up is needed in the presented patient to address this cause.

The recommended daily hydrocortisone dose in pediatric patients with PAI is 7–12 mg/m^2^, with a starting dose of 8 mg/m^2^, which is given in three divided doses [14,25,26]. In children with CAH, daily doses from 10 to 15 mg/m^2^ are recommended to adequately suppress adrenal androgen production [25,26]. In our patients, the average dose of hydrocortisone slightly exceeded the recommended range (16.6 mg/m^2^ in patients 1 and 2 and 13 mg/m^2^ in patient 3), which suggests that the production of glucocorticoids in patients with pathological *NNT* variants is greatly reduced. Other authors report that an NNT-deficient patient was started on a daily dose of 18 mg/m^2^ and continued through follow-up with a dose of 15 mg/m^2^ [20].

Corticosteroids, even at physiological doses, can affect BMD [26]. In the adult population, long-term follow-up of patients with Addison disease showed that patients as a group did not exhibit accelerated bone loss, however, among these, around 50% met osteoporotic criteria, which suggests that some patients are more susceptible to bone loss than others [27]. The observed low BMD in patient 1 could be due to the hydrocortisone supplementation [26], low BMI (below 5th percentile) [28], or perhaps oxidative stress [29]—although, we should state that we did not evaluate for other genetic causes of low BMD. Considering the role of NNT in maintaining the high GSH/GSSG ratio required for ROS detoxification, loss of NNT activity could result in increased oxidative stress [9,11,30]. ROS themselves can negatively affect bone as they increase the bone resorption process and inhibit osteogenesis and mineralization [29,31]. The negative effect of oxidative stress on bone turnover has already been described in children with type 1 diabetes mellitus [31]. To the best of our knowledge, we are the first to report the possible association between *NNT*-caused oxidative stress and bone formation, which should be confirmed on a larger cohort of NNT deficient patients.

In addition to the impairment of adrenal glands, unusual pubertal development was observed in the grown-up siblings. Our two patients progressed into puberty at the normal age and had no morphological changes in testicular parenchyma. Hypophysis was responsive to GnRH stimulation, excluding a possible gonadotropin deficiency. The testosterone and inhibin B levels were normal, while low–normal levels of adrenal androgens could be the result of adrenal tissue impairment associated with NNT deficiency. Their testicular volume (15 mL) rose slowly and was, during the last visit, at the lower level of normal. The delayed correction of one-sided cryptorchidism itself could cause damage to the affected testicle, thereby resulting in lower testicular volume; however, both grown-up siblings had similar development for testicular volume, although only one had one-sided testicular retention. Furthermore, previous research indicates that *NNT* is also expressed in the testes [12]. While pubertal development was slower in both patients, even in the presence of low–normal testicular volume, we are not convinced that both achieved their maximal potential and further follow-up is necessary. Only one report of azoospermia in NNT-deficient patients exists to date, and other authors also described testosterone-producing testicular tumors and peripheral precocious puberty [9,20]. These findings pose a question about whether NNT deficiency also affects pubertal development and gonadal function.

The heart is an energy demanding organ with vigorous cellular respiration and NNT defects have been associated with cardiomyopathies [32]. In mouse models, while NNT ablation by itself was not causative of cardiomyopathy [33], a functional copy of *NNT* was shown to prolong survival during gestation and to preserve cardiac function in sub-strains with defective superoxide dismutase [34], while the suppression of *NNT* led to ventricular dysfunction [35]. Furthermore, the decreased activity of NNT was observed in the failing human heart [36]. In NNT-defective patients, hypertrophic cardiomyopathy was already described by Roucher-Boulez et al. [9], however this finding could be accidental and caused by an unidentified gene defect. By performing ECG and transthoracic echo, we could not find signs suggestive of cardiomyopathy or heart failure in our patients, however, continued follow-up is warranted. To conclude, cardiac disease is not commonly described in these patients and NNT function loss may not even be responsible for cardiac manifestations, however this could also be due to the short follow-up period or to an absence of targeted diagnostic procedures [21]. More data on the subject is needed.

In conclusion, a novel frameshift variant in the *NNT* gene was identified in a non-consanguineous family from Slovenia. All three homozygous brothers developed isolated glucocorticoid deficiency. As we found that cortisol deficiency seems to be a gradually progressing condition, early hormonal testing might not rule out this condition. Genetic testing proved efficient in the early identification of the disease in siblings and prevented the development of a life-threatening Addisonian crisis. Slower than usual spontaneous puberty was also observed in the grown-up siblings, who achieved low–normal testicular volume without apparent gonadal dysfunction despite varicocele, which was treated in both siblings, and delayed treatment of cryptorchidism in one sibling. BMD was in the range for osteopenia in two patients, which could be caused by hydrocortisone alone or by the oxidative stress from NNT deficiency.

## Figures and Tables

**Table 1:** **GC-MS urinary steroid metabolome analysis in patient 3.** Spot urine was collected at the age of 6 months prior to hydrocortisone treatment initiation. Abnormal results are indicated by *.

Steroid Abbreviation	Steroid Name	Value (μg/L)	Reference Range (μg/L)
A5-3b,17a	5-androstene-3β,17α-diol	13	0–40
DHEA	5-androstene-3β-ol-17-on (dehydroepiandrosterone)	0	0–0
A5-3b,17b	5-androstene-3β,17β-diol	0	0–0
11-O-An	5α-androstane-3α-ol-11,17-dione (11-oxo-androsterone)	20	0–40
Po-5b,3a	5β-pregnane-3α,17α-diol-20-one (17a-OH-pregnanolone)	11	10–80
11-OH-An	5α-androstane-3α,11β-diol-17-one (11-hydroxy-androsterone)	22	20–70
11-OH-Et	5β-androstane-3α,11β-diol-17-one (11-hydroxy-etiocholanolone)	0	0–40
Po-5a,3a	5α-pregnane-3α,17α-diol-20-one	8	5–50
16a-OH-DHEA	5-androstene-3β,16α-diol-17-one	548	0–750
PD	5β-pregnane-3α,20α-diol (pregnanediol)	0	0–250
PT	5β-pregnane-3α,17α,20α-triol (pregnanetriol)	6	0–105
P5D	5-pregnene-3β,20α-diol (pregnenediol)	62	0–75
A5T-16a	5-androstene-3β,16α,17β-triol (androstenetriol-16α)	439	0–480
THS	5β-pregnane-3α,17α,21-triol-20-one (tetrahydro-11-deoxycortisol)	168	33–280
11-O-PT	5β-pregnane-3α,17α,20α-triol-11-one (11-oxo-pregnanetriol)	0	0–0
P5T-17a	5-pregnene-3β,17α,20α-triol (pregnenetriol-17α)	40	0–140
THE	5β-pregnane-3α,17 α,21-triol-11,20-dione (tetrahydro-cortisone)	869	465–1570
THA	5β-pregnane-3α,21-diol-11,20-dione	36	0–230
THB	5β-pregnane-3α,11β,21-triol-20-one (tetrahydro-corticosteron)	0	0–250
a-THB	5α-pregnane-3α,11β,21-triol-20-one (allo-tetrahydro-corticosteron)	92	0–100
THF	5β-pregnane-3α,11β,17α,21-tetrol-20-one (tetrahydro-cortisol)	122	10–200
a-THF	5α-pregnane-3α,11β,17α,21-tetrol-20-one (allo-tetrahydro-cortisol)	629	10–1000
a-Cl	5β-pregnane-3α,17α,20α,21-tetrol-11-one (α-Cortolone)	184	113–350
b-C	5β-pregnane-3a,11b,17a,20β,21-pentol (β-Cortol)	40	10–100
b-Cl	5β-pregnane-3α,17α,20β,21-tetrol-11-one (β-Cortolone)	182	30–800
a-C	5β-pregnane-3α,11β,17α,20α,21-pentol (α-Cortol)	0 *	150–525
F	4-pregnene-11β,17α,21-triol-3,20-dione (cortisol)	58	20–100
6b-OH F	4-pregnene-6β,11β,17α,21-tetrol-3,20-dione (6β-hydroxycortisol)	110	0–660
20a-DHF	4-pregnene-11β,17α,20α,21-tetrol-3-one (20α-dihydrocortisol)	28	0–100

## Data Availability

The data presented in this study are openly available in Mendeley Data at doi: 10.17632/zgytmbrnr2.1. Direct URL to data: https://data.mendeley.com/datasets/zgytmbrnr2/1 (accessed on 23 March 2022).
